# Oral rehydration salts, zinc supplement and rota virus vaccine in the management of childhood acute diarrhea

**DOI:** 10.4103/1319-1683.71988

**Published:** 2010

**Authors:** Abdulwahab MA Telmesani

**Affiliations:** *Department of Pediatrics, College of Medicine, Umm Al-Qura University, Makkah, Kingdom of Saudi Arabia*

**Keywords:** Diarrhea, childhood, oral rehydration salts, zinc, rota vaccine

## Abstract

Acute diarrhea remains a major cause of morbidity and mortality in children. Since the introduction of oral rehydration salts (ORS) mortality has dropped to less than 50% worldwide. Low osmolarity ORS improved the outcome and reduced the hospitalization further. Zinc difficiency has been found to be associated with severe episodes of acute diarrhea. Zinc supplement in developing countries did reduce the incidence and prevalence of diarrhea. In addition, Zinc supplement significantly reduced the severity of diarrhea and duration of the episode. In the Americas and Europe, Rota virus vaccine was 90% effective in preventing severe episodes of severe rotavirus gastroenteritis. This review concludes that low osmolarilty ORS, zinc supplementation and rotavirus vaccine are major factors in reducing the morbidity, mortality and hospitalization resulting from to acute gastroenteritis in childhood.

## INTRODUCTION

Acute diarrhea remains a leading cause of childhood deaths despite the undeniable success of oral rehydration therapy (ORT) over the years.[1] Since 1978, when the World Health Organization (WHO) and the United Nations Children’s Fund (UNICEF) adopted ORT with oral rehydration salts (ORS) solution as the primary tool for fighting dehydration, the mortality rate of children under the age of five suffering from acute diarrhea has fallen from 4.5 million to 1.8 million annually. However, in spite of this impressive achievement, acute diarrhea remains a leading cause of death in children in developing countries.[2] Worldwide, 3-5 billion cases of acute diarrhea occur each year in children under 5 years. While global mortality may be declining, the overall incidence of diarrhea remains unchanged at about 3.2 episodes per child year.[3] Two main dangers of diarrhea are death and malnutrition. Death from acute diarrhea is most often caused by the loss of a large amount of water and salt from the body leading to severe dehydration.[4] Much has been done to lessen severity, shorten the duration and to prevent the occurrence of diarrhea,[5] but the three most important achievements in this field are low osmolarity ORS, zinc supplementation and Rota virus vaccine.

## LOW OSMOLARITY ORAL REHYDRATION SALTS

After 20 years of research to improve ORS, a new formula has been developed and is recommended by WHO and UNICEF.[6] The efficacy of ORS solution for the treatment of children with acute non-cholera diarrhea has improved with the reduction of its sodium concentration to 75 mEq/l, its glucose concentration to 75 mmol/l, and its total osmolarity to 245 mOsm/l [Table 1]. The need for unscheduled supplemental intravenous therapy in children given this solution has reduced by 33%, stool output has reduced by about 20% and the incidence of vomiting by about 30%.[7] In a large multicenter trial of children with acute diarrhea not due to cholera, 675 children aged 1 to 24 months from 5 countries were randomized to receive standard or reduced osmolarity ORS. Although stool output and vomiting were not statistically different between the groups, the use of unscheduled intravenous fluids following initial rehydration was reduced in the group receiving reduced-osmolarity ORS (10% vs 15%) (odds ratio, 0.6; 95% confidence interval, 0.4-1.0). The occurrence of hyponatremia was not statistically different between the groups (11% in the reduced-osmolarity group vs 9% in the standard group) (odds ratio, 1.3; 95% confidence interval, 0.2-2.2).[9] In a meta-analysis of nine trials for the primary outcome, reduced osmolarity rehydration solution was associated with fewer unscheduled intravenous infusions compared with standard WHO rehydration solution (odds ratio 0.61, 95% confidence interval 0.47 to 0.81). Three trials reported that no patients required unscheduled intravenous infusion. Trials reporting secondary outcomes suggested that in the reduced osmolarity rehydration solution group, stool output was lower (standardized mean difference in the log scale - 0.214 (95% confidence interval - 0.305 to - 0.123; 13 trials) and there was less frequent vomiting (odds ratio 0.71, 0.55 to 0.92; six trials). Six trials sought the presence of hyponatraemia, with events in three studies, but there was no significant difference between the two arms.[10]

**Table 1:** ORS solutions developed by WHO[8]

Solution ORS	Glucose mmol/l	Na mmol/l	K mmol/l	Cl mmol/l	Base mmol/l	Osmolarity mosm/l
WHO 2005	75	75	20	65	10	245
WHO 2002	75	75	20	65	30	245
WHO 1975	111	90	20	80	30	311

## ZINC SUPPLEMENTATION

Diarrhea with severe zinc (Zn) deficiency has been observed in children in developing countries.[11] These findings prompted studies of Zinc supplementation in children with diarrhea. Thus, there is a compelling body of clinical data that Zn is likely to be effective both in the treatment of acute diarrhea and in its prophylaxis. The results of studies on zinc treatment as an adjunct in acute diarrhea have been reviewed in many studies.[12–14] These randomized controlled trials using doses of zinc ranging from 10 to 30 mg per day were conducted in children aged between 6 months and 3 years. Zinc supplemented children had 15% faster recovery (95% CI 4% to 24%) with a 22% reduction (95% CI 9% to 34%) in the odds of acute episodes lasting more than seven days. Subsequent trials show results consistent with the meta analysis.[14] One study was of major interest as it measured the impact on stool output, the most objective marker of severity and a useful proxy indicator for risk of dehydration, in 286 hospitalized children with acute diarrhea and dehydration.[15] In the zinc treated children, the total stool output was reduced by 31% (95% CI 1% to 52%) than in the placebo group. All studies showed that the effect of zinc did not vary significantly with age, or the nutritional status assessed by anthropometry. The effects were not dependent upon the type of zinc salts used: zinc sulfate, zinc acetate or zinc gluconate. There was little gain in efficacy when the commonly used 20 mg daily dose of elemental zinc was increased to 30-40 mg daily.[12–14]

A pooled analysis of randomized, controlled trials of zinc supplementation performed in nine low-income countries in Latin America and the Caribbean, South and Southeast Asia, and the Western Pacific, demonstrated that supplemental zinc led to an 18% reduction in the incidence of diarrhea and a 25% reduction in the prevalence of diarrhea.^16^ While the pooled analysis did not find differences in the effect of zinc by age, baseline serum zinc status, presence of wasting, or sex, the relevance of zinc supplementation to children in various geographic regions of the world remained unclear. Studies from Africa using zinc supplementation in 685 young children indicate significant benefit in the burden of diarrhea indicating that its effect may be consistent across various parts of the world,[17] even when administered with oral rehydration solution.[14] Recent studies in which zinc was used in the treatment of diarrhea in a community setting in Bangladesh (8070 children) also demonstrated a substantial reduction in concomitant use of antibiotics by health-care providers.[18] This suggests that there may be additional benefits with the use of zinc in the treatment of diarrhea.

Several changes have been found in Zn deficiency-associated diarrhea including morphologic changes in the intestine (e.g., villous atrophy, decreased brush-border activity, and altered intestinal permeability) and impairments of immune function (e.g., lymphoid tissue atrophy, reduction in lymphocyte count and T-helper cell proportion, cytotoxic activity of lymphocytes, and natural killer cell activity resulting in enhanced secretory response to cholera toxin.[19]

Despite several clinical observations of the efficacy of Zn in the treatment of acute diarrhea, the mechanism(s) by which Zn acts as an anti-diarrheal agent are poorly understood.[20] All of these successful clinical studies concluded that the possible mechanism for the beneficial effect of Zn on the duration of diarrhea included the following: (1) improved absorption of water and electrolytes by the intestine (undefined mechanism), (2) faster regeneration of gut epithelium,[21] (3) increased levels of enterocyte brush border enzymes[22] and/or (4) an enhanced immune response[23] leading to increased clearance of the pathogen(s) responsible for diarrhea from the intestine.[24]

A recent publication established that Zn inhibits cyclic adenosine monophosphate (cAMP)-induced complement one (Cl) secretion by inhibiting basolateral potassium (K) channels.[25] This study also showed the specificity of Zn to cAMP-activated Channels because Zn did not block calcium (Ca)-mediated K channels. Since this study was not performed in Zn-deficient animals, it provided evidence that Zn is likely to be effective in the absence of Zn deficiency. Another report recently provided evidence that Zn inhibits cholera toxin–induced, but not Escherichia coli heat-stable enterotoxin–induced ion secretion in cultured Caco-2 cells.[26]

Several other studies of potential action of Zn in cultured cell lines have been reported.[27,28] One such report showed that micro molar concentration of extra cellular Zn triggered a massive release of calcium from intracellular pools in the colonocytic cell line.^27^ If this observation is confirmed, one could argue that this sustained increase in intracellular Ca levels would enhance K efflux, leading to a hyperpolarization of cell membrane potential and the establishment of a favorable electrical gradient for Cl secretion.

Recently, WHO recommended that zinc supplementation should be provided at a dose of 10–20 mg per day for 10-14 days, and that this was efficacious in significantly reducing severity of diarrhea and duration of the episode.[29]

Zn is safe, well accepted, easily administered, and inexpensive. Most importantly, however, more laboratory-based studies that provide an understanding of the mechanism(s) whereby Zn is effective in the treatment of diarrhea are necessary.[20]

## ROTA VIRUS VACCINES

Rotavirus is a leading cause of severe, acute diarrhea in infants and young children throughout the world, and is responsible for an estimated 527,000 deaths among children under the age of 5 years each year. More than 90% of childhood deaths attributed to rotavirus infection occur in developing countries, making prevention by vaccination a priority in such settings.[30] Available rotavirus vaccines are summarized in Table 2.

**Table 2:** Live attenuated oral rotavirus vaccine[31]

Vaccine	Concept
LLR	Monovalent lamb strain (P[12]G10)
Rotateq	WC-3 based multivalent human-bovine reassortant
Rotarix(89-12)	Monovalent human strain (P[8]G1)
UK-reassortant vaccine	UK-based multivalent human–bovine reassortant
RV3	Neonatal strain (P[6]G3)
116E	Neonatal strain (P[11]G9)
1321	Neonatal strain (P[11]G10)

Large safety and efficacy trials conducted predominantly in the Americas and Europe demonstrated that both Rotateq and Rtoarix vaccines were highly (> 90%) efficacious in preventing severe rotavirus gastroenteritis. Neither vaccine was associated with intussusceptions.[32] While many countries quickly adopted a universal rotavirus vaccination policy, the absence of efficacy data from Africa and Asia precluded a recommendation for routine vaccination in those regions. The WHO, therefore, requested the testing of both vaccines in representative populations across both continents. In October 2005, a Phase III, placebo-controlled multi-centre clinical trial of Rotarix vaccine was initiated in South Africa and subsequently in Malawi. While the efficacy of the vaccine against severe rotavirus gastroenteritis was lower in Malawi than was observed in South Africa (77%), the public health impact of vaccination was likely to be great in Malawi because of the high incidence of severe rotavirus disease recognized during the trial.[33,34]

Recently, The World Health Organization recommended that rotavirus vaccination be included in all national immunization programs in order to provide protection against the virus.[35]

Finally, low osmolarilty ORS, zinc supplementation and rotavirus vaccine are major steps on the road to decreasing the morbidity and mortality of acute diarrhea which is considered one of the most serious diseases affecting infants and children under five years.
